# Corneal confocal microscopy demonstrates axonal loss in different courses of multiple sclerosis

**DOI:** 10.1038/s41598-021-01226-1

**Published:** 2021-11-04

**Authors:** Ioannis N. Petropoulos, Kathryn C. Fitzgerald, Jonathan Oakley, Georgios Ponirakis, Adnan Khan, Hoda Gad, Pooja George, Dirk Deleu, Beatriz G. Canibano, Naveed Akhtar, Ashfaq Shuaib, Ahmed Own, Taimur Malik, Daniel B. Russakoff, Joseph L. Mankowski, Stuti L. Misra, Charles N. J. McGhee, Peter Calabresi, Shiv Saidha, Saadat Kamran, Rayaz A. Malik

**Affiliations:** 1 Research Division, Qatar Foundation, Weill Cornell Medicine-Qatar of Cornell University, PO Box 24144, Education City, Doha, Qatar; 2 Department of Neurology, Johns Hopkins University School of Medicine, Baltimore, MD USA; 3 Voxeleron LLC, San Francisco, CA USA; 4 Neuroscience Institute, Hamad General Hospital, Doha, Qatar; 5 Department of Medicine, University of Alberta, Edmonton, AB Canada; 6 Department of Molecular and Comparative Pathobiology, Johns Hopkins University School of Medicine, Baltimore, MD USA; 7 Department of Ophthalmology, New Zealand National Eye Centre, University of Auckland, Auckland, New Zealand

**Keywords:** Diagnostic markers, Biomarkers, Neurology

## Abstract

Axonal loss is the main determinant of disease progression in multiple sclerosis (MS). This study aimed to assess the utility of corneal confocal microscopy (CCM) in detecting corneal axonal loss in different courses of MS. The results were confirmed by two independent segmentation methods. 72 subjects (144 eyes) [(clinically isolated syndrome (n = 9); relapsing–remitting MS (n = 20); secondary-progressive MS (n = 22); and age-matched, healthy controls (n = 21)] underwent CCM and assessment of their disability status. Two independent algorithms (ACCMetrics; and Voxeleron deepNerve) were used to quantify corneal nerve fiber density (CNFD) (ACCMetrics only), corneal nerve fiber length (CNFL) and corneal nerve fractal dimension (CNFrD). Data are expressed as mean ± standard deviation with 95% confidence interval (CI). Compared to controls, patients with MS had significantly lower CNFD (34.76 ± 5.57 vs. 19.85 ± 6.75 fibers/mm^2^, 95% CI − 18.24 to − 11.59, *P* < .0001), CNFL [for ACCMetrics: 19.75 ± 2.39 vs. 12.40 ± 3.30 mm/mm^2^, 95% CI − 8.94 to − 5.77, *P* < .0001; for deepNerve: 21.98 ± 2.76 vs. 14.40 ± 4.17 mm/mm^2^, 95% CI − 9.55 to − 5.6, *P* < .0001] and CNFrD [for ACCMetrics: 1.52 ± 0.02 vs. 1.45 ± 0.04, 95% CI − 0.09 to − 0.05, *P* < .0001; for deepNerve: 1.29 ± 0.03 vs. 1.19 ± 0.07, 95% − 0.13 to − 0.07, *P* < .0001]. Corneal nerve parameters were comparably reduced in different courses of MS. There was excellent reproducibility between the algorithms. Significant corneal axonal loss is detected in different courses of MS including patients with clinically isolated syndrome.

## Introduction

Multiple Sclerosis (MS) is characterized by inflammation and neurodegeneration with cumulative axonal loss being the main determinant of disease progression^1^. However, the accurate quantification of axonal loss to help predict patient outcomes and assess therapeutic benefit in trials of neuroprotection is a major challenge. Previous studies have established that retinal optical coherence tomography^2^ along with non-conventional magnetic resonance imaging techniques^3^ such as brain volumetric analysis, diffusion tensor imaging, magnetization transfer imaging and proton magnetic resonance spectroscopy may act as potential surrogate markers of neurodegeneration in MS.

A substantial body of evidence suggests that imaging of the corneal sub-basal nerve plexus using corneal confocal microscopy (CCM) is a sensitive method to diagnose and stratify the severity of diabetic^4^ and other peripheral neuropathies^5,6^. CCM is a well-tolerated technique with high intra- and inter-operator reproducibility^7^. Corneal neurodegeneration is related to clinical measures of neuropathy^8^ and has shown comparable diagnostic performance to intraepidermal nerve fiber loss in diabetic neuropathy^9^. It occurs early in patients with sub-clinical neuropathy^10^ and predicts the development of clinically established diabetic neuropathy^11^. Altered corneal nerve pattern complexity has been established in patients with neuropathy and can be quantified by corneal nerve fractal dimension (CNFrD) analysis^12^. Furthermore, a recent phase 2 clinical trial^13^ of Cibinetide™ (ARA290; helix B surface peptide) in patients with sarcoidosis-associated neuropathy demonstrated a significant improvement in corneal nerve fiber length (CNFL) and intra-epidermal nerve fiber length. Simultaneous pancreas and kidney transplantation in patients with type 1 diabetes results in an improvement in CNFL within 6 months^14^ which is followed by an improvement in neuropathic symptoms and neurophysiology at 36 months^15^.

More recently, studies using CCM have shown significant corneal axonal loss in patients with Parkinson’s disease^16,17^ and dementia^18^. Studies of smaller patient cohorts with predominantly relapsing–remitting MS (RRMS) have also demonstrated corneal axonal loss and related it to clinical disability^19–23^, retinal nerve fiber layer thinning^22^ and an increase in corneal immune cell density^19,24^. It is not known if corneal axonal loss occurs in patients with clinically isolated syndrome (CIS) and whether it differs from RRMS and secondary progressive MS (SPMS). The relevance of corneal nerve loss in MS may be challenged. However, the corneal subbasal nerve plexus is comprised of unmyelinated sensory nerve fibers derived from pseudo-unipolar neurons located in the trigeminal ganglion, which also project centrally into the brainstem; and trigeminal lesions have been demonstrated in-vivo^25^ and in pathological specimens^26^ from patients with MS. These findings argue that corneal nerve loss may act as a surrogate marker for central neurodegeneration and underpin the potential of CCM as a rapid, non-invasive surrogate marker for neurodegeneration in MS.

Previously, corneal nerve morphology has been evaluated by undertaking manual quantification. However, manual analysis is time-consuming and subjective with a risk of bias. To overcome this challenge, we have developed an automated CCM image segmentation algorithm based on machine-learning (ACCMetrics)^27^ and validated^4^ it in patients with diabetic neuropathy. A recent study in a model of human immunodeficiency virus-associated neuropathy^28^ has described a novel CCM image segmentation algorithm^29^ based on deep learning (Voxeleron deepNerve). There are significant differences in how these methods operate. Traditional machine learning requires a set of predefined criteria for pixel detection without spatial context. Deep learning detects features through a series of image transformations while maintaining the spatial relationship of neighboring pixels. Subsequently, this information is backpropagated to facilitate learning. Its performance is directed by a loss function, which determines output accuracy in relation to input data. The present study aimed to compare corneal axonal loss in different courses of MS including CIS using two independent, objective image segmentation algorithms.

## Results

Data are expressed as mean ± standard deviation with 95% confidence interval (CI). Amongst patients with MS, n = 35 (69%) were females, and their mean age was 37.11 ± 9.55 years. Amongst healthy control participants, n = 9 (42%) were females, and the mean age was 39.0 ± 10.23 years. There was no difference in age between healthy controls compared to the MS group as a whole (95% CI − 7.06 to 3.1, *P* = 0.43) or compared to CIS (vs. 36.33 ± 6.86 years, 95% CI − 8.18 to 12.76, *P* = 0.99); RRMS (vs. 33.85 ± 8.65 years, 95% CI − 3.44 to 12.98, *P* = 0.54), and SPMS (vs. 40.18 ± 10.75 years, 95% CI − 9.58 to 6.45, *P* = 0.99]. Disease duration was significantly longer in SPMS (9.73 ± 3.74 years, 95% CI − 10.58 to − 3.98, *P* < 0.0001) and RRMS (8.15 ± 3.71 years, 95% CI − 9.05 to − 2.36, P = 0.0004) compared to CIS (2.44 ± 1.33 years, 95% CI 1.42–3.47). Patients with SPMS had a significantly higher expanded disability status scale (EDSS) score compared to CIS (4.09 ± 2.29 vs. 0.67 ± 0.66, 95% CI − 5.0 to − 1.8, *P* < 0.0001) and RRMS (vs. 0.88 ± 0.99, 95% CI − 4.49 to − 1.95, *P* < 0.0001) with no significant difference between CIS and RRMS (95% CI − 1.86 to 1.44, *P* = 0.98). Patients with SPMS had a significantly higher number of relapses compared to CIS (3.70 ± 2.41 vs. 0, 95% CI − 5.49 to − 1.91, *P* < 0.0001) and RRMS (vs. 1.70 ± 1.42, 95% CI − 3.41 to − 0.59, *P* < 0.003) with no significant difference between CIS and RRMS (95% CI − 3.49 to 0.08, *P* = 0.06). A description of the cohort is provided in Table 1.

**Table 1:** Demographic and clinical characteristics of the study cohort.

	Controls	CIS	RRMS	SPMS
*N*	21	9	20	22
Age	38.62 ± 10.16	36.4 ± 6.24	34.03 ± 8.2	40.2 ± 10.58
Disease duration (years)	–	2.44 ± 1.33	8.15 ± 3.72	9.73 ± 3.74
Sex (% female)	47.6	66.6	75	63.6
Ethnicity (White/Black/South Asian)	16/0/5	6/1/2	16/2/2	18/0/4
ON history (%)	–	44.4	60.0	63.6
EDSS	–	0.67 ± 0.66	0.88 ± 0.98	4.09 ± 2.29

Data are expressed as mean ± standard deviation or as %. *CIS* clinically Isolated syndrome, *EDSS* expanded disability status scale, *ON* optic neuritis, *RRMS* relapsing remitting multiple sclerosis, *SPMS* secondary progressive multiple sclerosis.

### ACCMetrics

**Figure 1:**
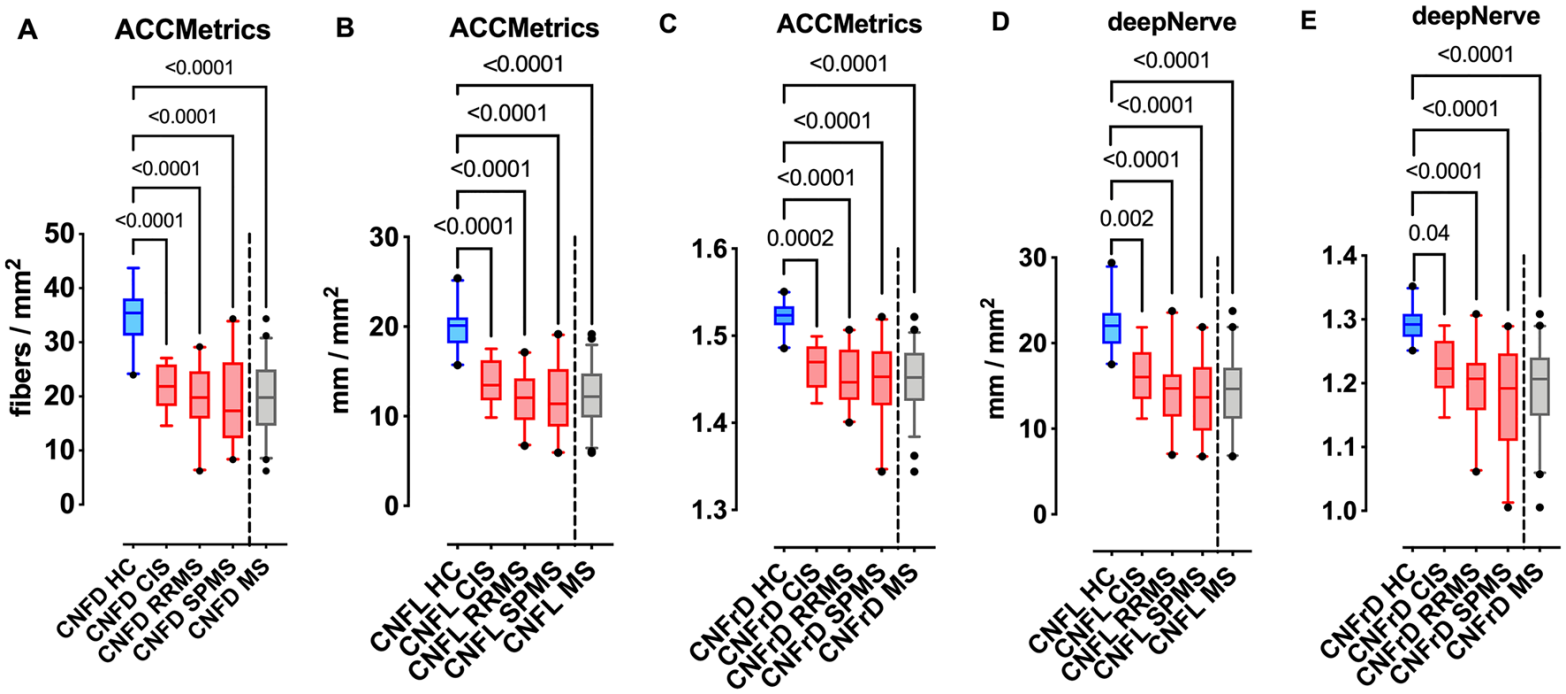
Graphs represent box (mean and interquartile range) and whiskers (5th and 95th percentile) plots with outliers (solid black dots). Blue color corresponds to HC, red to different MS courses and grey to the whole MS group. Graphs (**A**) CNFD, (**B**) CNFL and (**C**) CNFrD correspond to classification performance as measured by ACCMetrics; and graphs (**D**) CNFL and (**E**) CNFrD correspond to Voxeleron deepNerve.

In patients with MS compared to healthy controls, there was a significant reduction in corneal nerve fiber density (CNFD) (34.76 ± 5.57 vs. 19.85 ± 6.75, 95% CI − 18.24 to − 11.59, *P* < 0.0001); CNFL (19.75 ± 2.39 vs. 12.40 ± 3.30, 95% CI − 8.94 to − 5.77, *P* < 0.0001 and CNFrD (1.52 ± 0.02 vs. 1.45 ± 0.04, 95% CI − 0.09 to − 0.05, *P* < 0.0001). The difference remained significant compared to healthy controls in patients with CIS: CNFD (22.02 ± 4.35, 95% CI 5.74–19.72, *P* < 0.0001), CNFL (13.76 ± 2.62, 95% CI 2.69–9.3, *P* < 0.0001) and CNFrD (1.47 ± 0.03, 95% CI 0.02–0.09, *P* = 0.0002); RRMS: CNFD (19.48 ± 6.15, 95% CI 9.81 to 20.76, *P* < 0.0001), CNFL (12.14 ± 2.85, 95% CI 5.01 to 10.2, *P* < 0.0001) and CNFrD (1.45 ± 0.05, 95% CI 0.04–0.1, *P* < 0.0001], and SPMS: CNFD (19.29 ± 8.03, 95% CI 10.12–20.82, *P* < 0.0001], CNFL (12.08 ± 3.88, 95% CI 5.14–10.2, *P* < 0.0001) and CNFrD (1.45 ± 0.05, 95% CI 0.05–0.1, *P* < 0.0001). Patients with a prior history of optic neuritis (ON) compared to patients without ON (NON) showed a trend towards lower CNFD (18.98 ± 7.28, 95% CI 16.3–21.7 vs. 21.1 ± 5.85, 95% CI 18.4–23.7, *P* = 0.29); CNFL (11.96 ± 10.7, 95% CI 10.7–13.2 vs. 13.04 ± 11.5, 95% CI 11.5–14.6, *P* = 0.25); and CNFrD (1.45 ± 0.04, 95% CI 1.44–1.46 vs. 1.46 ± 0.04, 95% CI 1.44–1.48, *P* = 0.4) but the difference was not significant. There was no significant difference between different MS courses; consistent results were obtained after adjusting for age, sex, race, and history of ON. EDSS correlated significantly with CNFD (r = − 0.32, 95% CI − 0.55 to − 0.04, *P* = 0.02), CNFL (r = − 0.32, 95% CI − 0.52 to − 0.001, *P* = 0.04) and CNFrD (r = − 0.29, 95% CI − 0.54 to − 0.02, *P* = 0.03). The detailed results are presented in Table 2 and Fig. 1A–C.

**Table 2:** CCM image quantification with ACCMetrics and Voxeleron deepNerve.

Parameter	Controls	*Patients with MS*				*P* *value (vs. Controls)*			
		Total MS	CIS	RRMS	SPMS	MS	CIS	RRMS	SPMS
**ACCMetrics**									
CNFD (fibers/mm^2^)	34.76 ± 5.57	19.85 ± 6.75	22.02 ± 4.35	19.48 ± 6.15	19.29 ± 8.03	< .0001^a^	< .0001^b^	< .0001^b^	< .0001^b^
CNFL (mm/mm^2^)	19.75 ± 2.39	12.40 ± 3.30	13.76 ± 2.62	12.14 ± 2.85	12.08 ± 3.88	< .0001^a^	< .0001^b^	< .0001^b^	< .0001^b^
CNFrD	1.52 ± 0.02	1.45 ± 0.04	1.47 ± 0.03	1.45 ± 0.03	1.45 ± 0.05	< .0001^a^	.0002^b^	< .0001^b^	< .0001^b^
**Voxeleron deepNerve**									
CNFL (mm/mm^2^)	21.98 ± 2.76	14.40 ± 4.17	16.18 ± 3.39	14.34 ± 3.76	13.74 ± 4.73	< .0001^a^	.002^b^	< .0001^b^	< .0001^b^
CNFrD	1.29 ± 0.03	1.19 ± 0.07	1.23 ± 0.05	1.19 ± 0.06	1.18 ± 0.08	< .0001^a^	.04^b^	< .0001^b^	< .0001^b^

*CIS* clinically Isolated Syndrome, *RRMS* relapsing remitting multiple sclerosis, *SPMS* secondary progressive multiple sclerosis, *MS* multiple sclerosis, *CNFD* corneal nerve fiber density, *CFL* corneal nerve fiber length, *CNFrD* corneal nerve fractal dimension.

Data are expressed as mean ± standard deviation.

^a^Unpaired *t*-test.

^b^One-way analysis of variance.

### Voxeleron deepNerve

In patients with MS compared to healthy controls, there was a significant reduction in CNFL (21.98 ± 2.76 vs. 14.40 ± 4.17, 95% CI − 9.55 to − 5.6, *P* < 0.0001, unpaired t-test), and CNFrD (1.29 ± 0.03 vs. 1.19 ± 0.07, 95% CI − 0.13 to − 0.07, *P* < 0.0001). The difference remained significant compared to healthy controls in patients with CIS: CNFL (16.18 ± 3.39, 95% CI 1.81–9.8, *P* = 0.002) and CNFrD (1.23 ± 0.05, 95% CI 0.002–0.13, *P* = 0.04); RRMS: CNFL (14.34 ± 3.76, 95% CI 4.51–10.77, *P* < 0.0001) and CNFrD (1.19 ± 0.06, 95% CI 0.05–0.2, *P* < 0.0001); and SPMS: CNFL (13.74 ± 4.73, 95% CI 5.18–11.29, *P* < 0.0001) and CNFrD (1.18 ± 0.08, 95% CI 0.06–0.16, *P* < 0.0001). Patients with MS and ON compared to NON showed a trend towards lower CNFL (13.83 ± 3.99, 95% CI 12.3–15.3 vs. 15.23 ± 4.37, 95% CI 13.2–17.2, *P* = 0.24); and CNFrD (1.19 ± 0.06, 95% CI 1.16–1.21 vs. 1.2 ± 0.07, 95% CI 1.17–1.24, *P* = 0.45) but the difference was not significant. There was no difference between different MS courses; consistent results were obtained after adjusting for age, sex, race and history of ON. EDSS correlated significantly with CNFL (r = − 0.32, 95% CI − 0.55 to − 0.04, *P* = 0.02). The detailed results are presented in Table 2 and Fig. 1D and E.

### CCM image analysis comparison

**Figure 2:**
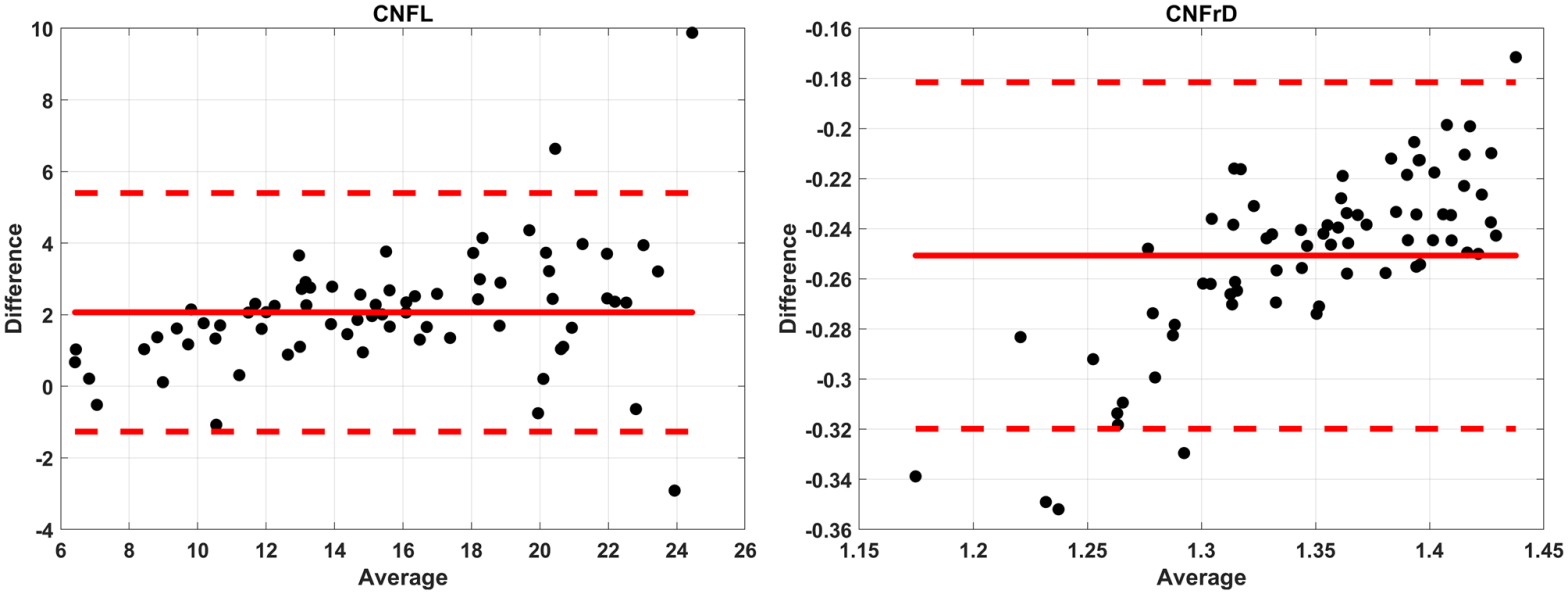
Bland–Altman (mean ± LOA) plots for (**A**) CNFL and (**B**) CNFrD as an indication of agreement between ACCMetrics and Voxeleron deepNerve.

There was a strong linear relationship between ACCMetrics and deepNerve for CNFL (r = 0.97, *P* < 0.0001) and CNFrD (r = 0.94, *P* < 0.0001). Spearman correlations were also consistent after adjusting for age, sex, race and history of ON and similar correlations were observed using Pearson correlations. The intra-class correlation co-efficient (ICC) was high for CNFL (ICC 0.82, 95% CI 0.74–0.87) and CNFrD (ICC 0.83, 95% CI 0.76–0.88) indicating excellent reproducibility. Bland–Altman analysis (Table 3 and Fig. 2A,B) showed that fully automated CCM image analysis performed by two independent segmentation methods on the same set of images has generally high agreement for measures of length and fractal analysis once we accounted for differences in the variability across levels of measurement (following approaches described by Bland and Altman^30^ for more complex associations between measures).

**Table 3:** Bland–Altman analysis: Voxeleron deepNerve versus ACCMetrics.

Parameter (n = 72)	Mean difference (95% CI)	Lower LOA	Upper LOA	Range
CNFL	− 2.07 (± 0.69)	− 5.40	1.27	6.66
CNFrD	− 0.25 (± 0.01)	− 0.32	− 0.18	0.14

*CI* confidence interval, *CNFL* corneal nerve fiber length, *CNFrD* corneal nerve fractal dimension, *LOA* limits of agreement.

## Discussion

Over the last two decades, CCM has emerged as a powerful surrogate marker of peripheral neuropathy^4–6^. We have shown that corneal nerve loss is related to the genotype, and neurological disability assessed using the Scale for the Assessment and Rating of Ataxia, Friedreich's Ataxia Rating Scale and quantitative gait assessment in patients with Friedreich’s ataxia^6^. More recent studies have shown that corneal axonal loss also occurs in central neurodegenerative conditions and is associated with the severity of neurological deficits^16,19–21^ and cognitive impairment^18^. Accurate quantification of corneal nerve alterations in CCM images is challenging due to the small image size, variable contrast and lack of universally accepted criteria to identify corneal nerve loss. Nevertheless, earlier studies^4,31^ have shown that both manual and automated measures of corneal nerve morphology (CNFD, CNFL, CNFrD) are a robust means to assess the severity of peripheral neuropathy. In this study, we have applied two independently validated automated segmentation algorithms^27,29^ on the same set of CCM images from patients with MS and healthy individuals.

The first finding in our study was a significant reduction in CNFD, CNFL and CNFrD in patients with CIS, RRMS and SPMS compared to healthy controls. Whilst these findings confirm the results of previous smaller studies showing corneal nerve loss in patients with predominantly RRMS^19–22^; we now additionally demonstrate significant corneal nerve loss in patients with CIS and SPMS. Indeed, previous studies have shown a comparable degree of retinal nerve fiber layer thinning in CIS^32^ and other courses of MS^33^. Moreover, corneal nerve loss was comparable between patients with RRMS and SPMS. Previous studies^19–22^ have reported an association between corneal nerve loss and disability, but not disease stage, suggesting that corneal nerve loss may occur early in MS. The corneal subbasal nerve axons are derived from the trigeminal ganglion but are part of the peripheral nervous system. Therefore, the relevance of corneal axonal loss to central neurodegeneration in MS may be questionable. However, all studies to date have consistently demonstrated corneal nerve loss and related it to neurological disability in different cohorts of patients with MS^19–23^. Indeed, there is evidence of substantial neurodegeneration in the spinal cord of patients with MS which contributes to significant disability^34^. Diffuse synaptic pathology in the grey matter has also been shown to selectively affect distal axonal density due to reduced axonal transport^35^. The lack of difference between different MS courses in the present study may reflect underlying differences in disease duration with relatively mild neurological disability, especially in patients with SPMS, and a small cohort size.

The second main finding in our study was that ACCMetrics and Voxeleron deepNerve measurements of corneal nerve loss were strongly associated, despite the two algorithms employing different underlying segmentation methods to quantify CCM images. ACCMetrics uses a trained feature detection model based on a set of predefined criteria for nerve fiber segmentation provided by a ground truth dataset. Voxeleron deepNerve^29^ applies a dense series of overlapping filters, pre-learned using a deep neural network architecture, to the original image, thereby generating a nerve probability image and the final delineation. Both algorithms have strengths; ACCMetrics has been validated in multiple studies^4,9,12^ and can measure additional metrics such as CNFD and corneal nerve branch density, which may provide an insight into nerve regeneration in clinical trials of disease modifying therapies^13,15,36^. On the other hand, Voxeleron deepNerve is capable of segmenting various types of corneal nerve images such as immunohistochemically stained whole corneal mounts^28^ and in-vivo CCM images and has an advantage when quantifying larger corneal nerve maps. Segmentation accuracy by deepNerve may also be less liable to image noise as a result of poor patient cooperation or less experienced examiners, resulting in sub-optimal image quality. This is because deep learning networks can detect image features while retaining their spatial relationship. They furthermore facilitate iterative learning by backpropagating this information into the network. This is relevant to corneal nerve fibers, which appear as sequences of neighboring nerve pixels against a dark background. Another advantage is that deep learning performance is directed by a loss function, which determines how accurate the final output is in relation to the input data, allowing a tradeoff between false positives and false negatives. Clearly, automation is a major strength of both methods as it minimizes bias, accelerates image quantification, and makes CCM more suitable for prospective and multi-center trials. Both techniques are subject to selection bias if the predefined criteria or training dataset respectively are not sufficiently inclusive. However, deep learning models may be superior to traditional machine learning methods due to their versatility.

The present study has some limitations. First, our image sampling approach may have introduced selection bias favoring lower mean values compared to the true mean of the central subbasal nerve plexus^37^ especially for patients with more advanced disability. Second, the results from this study cannot be generalized to all deep learning algorithms. As the field of artificial intelligence is constantly evolving, a system with a different network architecture may produce different results. Third, although no participant was clinically diagnosed with trigeminal neuralgia, trigeminal-related pathology may have contributed to the observed differences. Reassuringly, an earlier study^20^ found no difference in corneal nerve density in patients with and without trigeminal neuralgia-related symptoms. In summary, we have shown significant corneal axonal loss in different courses of MS and for the first time in patients with CIS using two independent image segmentation algorithms. These data urge the need for further prospective CCM studies in larger cohorts of patients with different courses of MS evaluating additional morphological features, such as the inferior whorl^38^, and Langerhans cells^19,24^ longitudinally and in relation to trigeminal neuralgia and therapeutic intervention.

## Methods

### Study subjects

**Figure 3:**
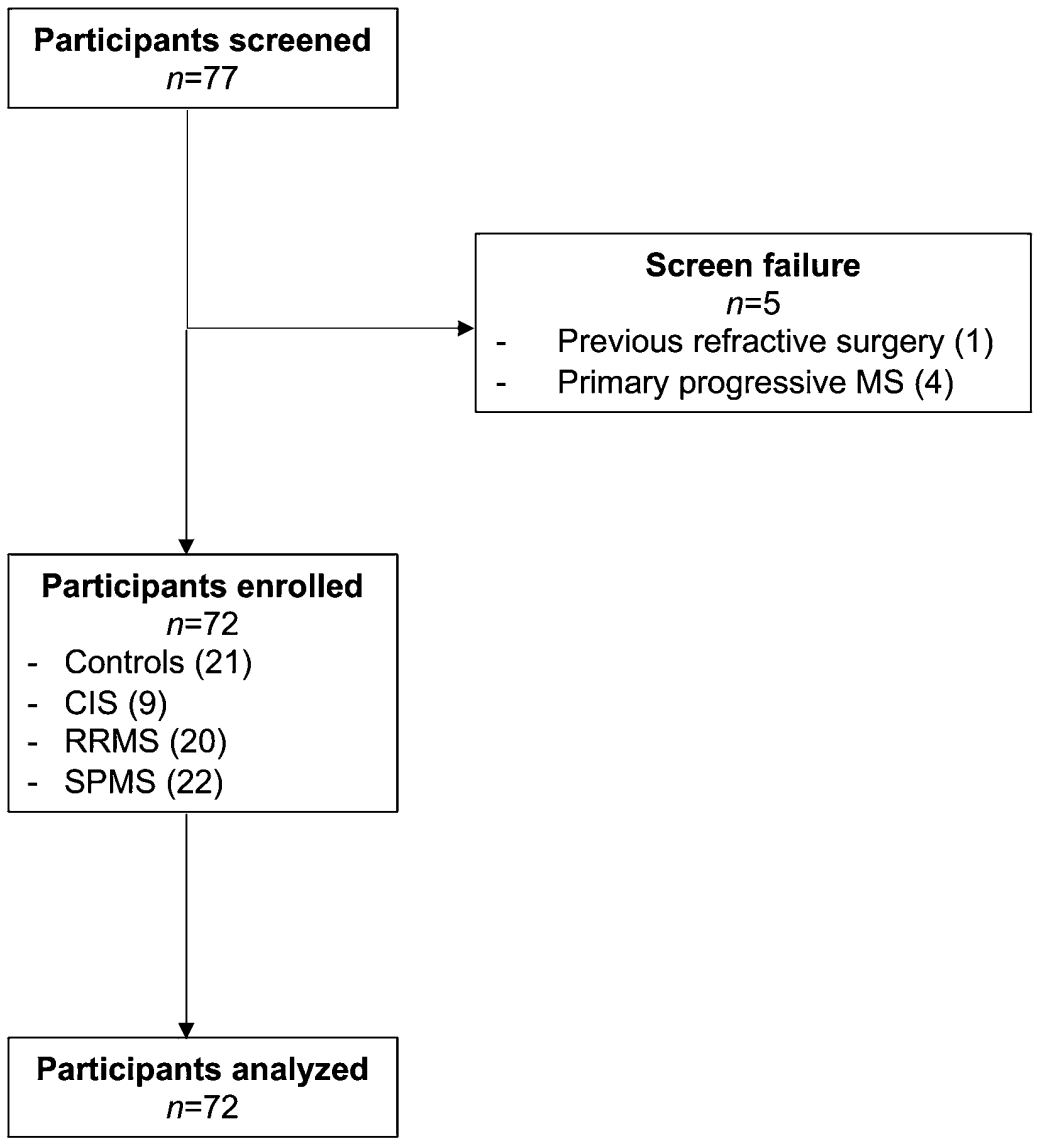
Flowchart of the study cohort.

This is a single-center, cross-sectional, observational study conducted between February 2017 and March 2018. Patients with CIS (n = 9), RRMS (n = 20) and SPMS (n = 22) attending the neurology outpatient department of Hamad General Hospital in Doha, Qatar, and age-matched, healthy controls (n = 21) were recruited (Fig. 3). Main outcome measures were CNFD, CNFL and CNFrD quantified by ACCMetrics and Voxeleron deepNerve respectively. This study adhered to the tenets of the declaration of Helsinki and obtained prospective approval from the institutional review board of Weill Cornell Medicine-Qatar (no. 15-00064). Informed, written consent for research was obtained from all subjects prior to participation. Reporting of results in this study followed the STROBE guidelines^39^. Inclusion criteria were diagnosis of CIS or MS based on the revised McDonald’s criteria (2010)^40^ and age 18–75 years. Patients with MS and healthy controls who were contact lens users, diagnosed with ophthalmic disease (e.g., glaucoma, vitreoretinal or corneal disorders), had active ON or had undergone refractive surgery were excluded. Patients with other metabolic, ophthalmologic, rheumatologic, or neurologic disorders that may cause neuropathy were excluded from participation in the study based on HbA1c, anti-nuclear antibody, serum B12/folate and immunoglobulins and a detailed medical history. All underlying anonymized data from the analysis presented in this manuscript are available for use on request to the corresponding author.

### Clinical and demographic information

Past medical history including ON history, disease duration and MS-associated relapses were obtained by a physician neurologist. The EDSS by Kurtzke^41^ was performed prior to CCM scans to rate neurological impairment in patients with MS. Briefly, the EDSS is a physician-administered composite for functional assessment of the central nervous system. It consists of an ordinal system ranging from 0 (normal neurological function) to 10 (death due to MS) in 0.5 increments (from EDSS > 1 onwards). Scores from 0 to 4 evaluate general neurological function, 4–6 focuses on walking ability and scores greater than 6 indicate loss of neurological independence.

### Corneal confocal microscopy

**Figure 4:**
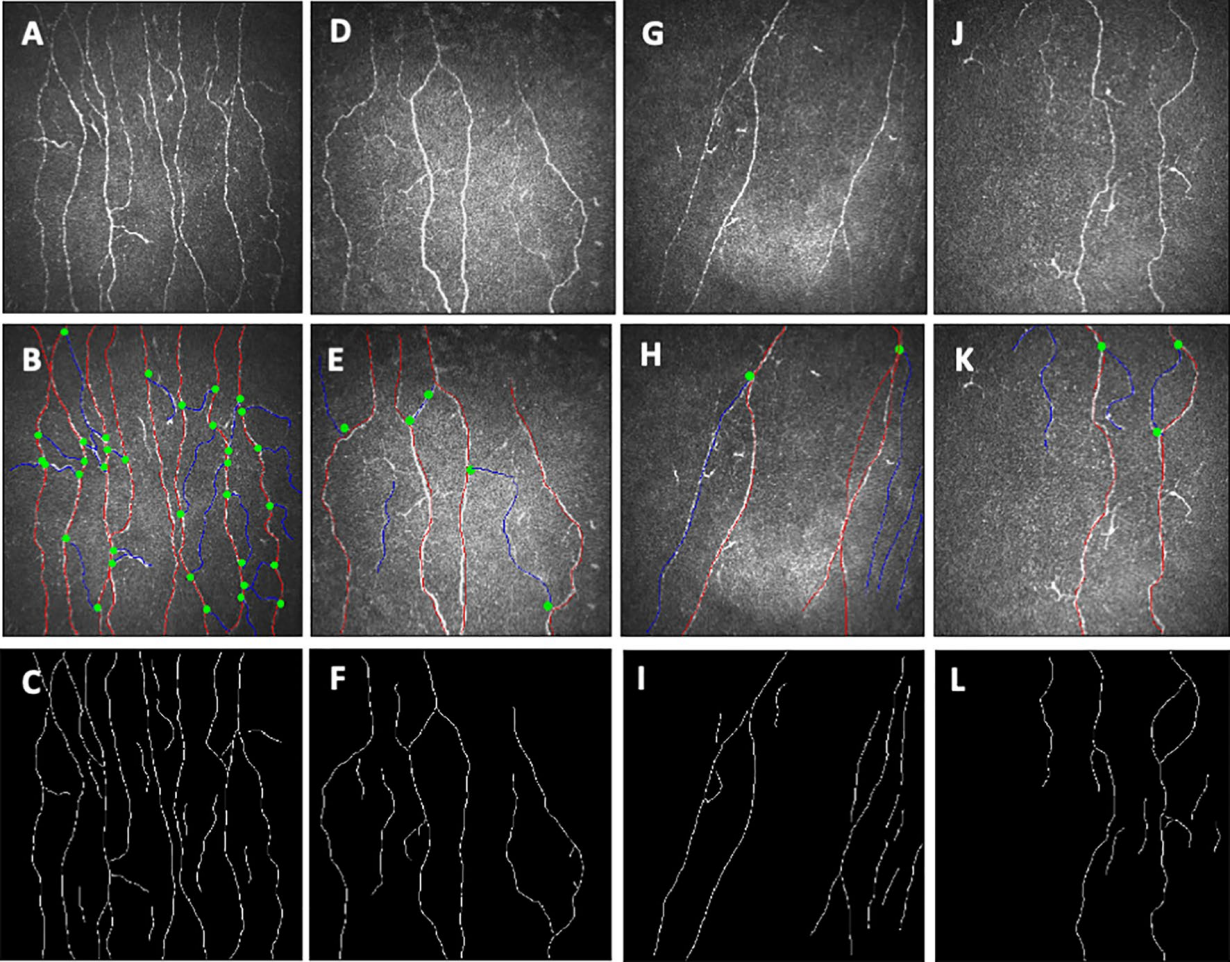
Row 1 (**A**,**D**,**G**,**J**): Original CCM images from a HC (**A**) and patients with CIS (**D**), RRMS (**G**) and SPMS (**J**) with identifiable corneal axonal loss. Row 2 (**B**,**E**,**H**,**K**): corresponding analyzed images with ACCMetrics to estimate CNFD (main fibers = red color), CNFL (fibers and branches = red and blue colors) and CNFrD. Row 3 (**C**,**F**,**I**,**L**): corresponding images analyzed with Voxeleron deepNerve.

All study participants underwent CCM (Heidelberg Retinal Tomograph III Rostock Cornea Module, Heidelberg Engineering GmbH, Heidelberg, Germany). This device uses a 670 nm wavelength helium neon diode laser, which is a class I laser and therefore does not pose any ocular safety hazard. A 63 × objective lens with a numerical aperture of 0.9 and a working distance, relative to the applanating cap (TomoCap^©^, Heidelberg Engineering GmbH, Heidelberg, Germany) of 0.0 to 3.0 mm is used. The size of each two-dimensional image produced is 384 μm × 384 μm with a 15° × 15° field of view and 10 μm/pixel transverse optical resolution. To perform the CCM examination, local anesthetic (0.4% benoxinate hydrochloride, Chauvin Pharmaceuticals, Chefaro, UK) was used to anaesthetize each eye and Viscotears (Carbomer 980, 0.2%, Novartis, UK) were used as the coupling agent between the cornea and the applanating cap. All subjects were asked to fixate on an outer fixation light throughout the CCM scan and an externally coupled camera was used to correctly position the applanating cap onto the central cornea. Images were acquired using the “section” mode on the Heidelberg eye explorer and the scanning duration for both eyes was 5–7 min. Based on depth, contrast and focus position 6 non-overlapping images/subject (3 out of 15 images per eye) from the central sub-basal nerve plexus were selected for analysis as per previously validated protocol^42^. In the present study, CCM results are presented as an average of all 6 images per patient. Investigators were masked to the MS course, disability severity and medication history during CCM examination and image selection was performed by a different investigator without knowledge of the participant. CCM image examples from a HC and patients with CIS, RRMS and SPMS are presented in Fig. 4.

### Image analysis

CCM images from both eyes (n = 448) were analyzed using two independent, purpose-designed CCM image segmentation algorithms: ACCMetrics (MA Dabbah, X Chen, J Graham & RA Malik, University of Manchester, Manchester, UK) and Voxeleron deepNerve (Dr. JL Mankowski, Johns Hopkins University School of Medicine, Baltimore, MD, USA; and Drs. JD Oakley and DB Russakoff, Voxeleron LLC, San Francisco, CA, USA hold a patent^43^ relating to aspects of the algorithm’s implementation) at the same time in different geographical locations. CCM image examples analyzed by ACCMetrics and Voxeleron deepNerve are presented in Fig. 4A,D,G,J.

#### ACCMetrics

ACCMetrics^27^ is a validated CCM image segmentation algorithm which measures CNFD (fibers/mm^2^)—the number of main nerve fibers per image divided by the area of the image, CNFL (mm/mm^2^)—the sum of the length of all nerves per image (main nerves + branches) and CNFrD—nerve fiber complexity per image. Briefly, automated corneal nerve fiber detection consists of two steps: (1) CCM image enhancement and nerve fiber detection and (2) quantification of the morphometric parameters. A dual-model feature descriptor combined with a neural network classifier was used to train the computer to distinguish nerve fibers from the background. In the quantification process, end points and branch points of the detected nerve fibers were used to construct a connectivity map and each segment was classified as a main nerve fiber or branch. CNFrD estimation is based on the detection of nerve fibers against the background using a machine-learning approach^12^, and measures nerve complexity as the ratio of the change in detail to change in scale using a box counting method. Analyzed image examples by ACCMetrics are shown in Fig. 4B,E,H,K.

#### Voxeleron deepNerve

This image segmentation algorithm termed deepNerve extends the earlier work of Dorsey et al.^28^ using a deep learning-based approach for the detection and analysis of corneal subbasal nerves^29^. This is a supervised learning approach where pixel wise segmentation was compared with manually traced data using NeuronJ^44^ in 60 images acquired from the University of Auckland^45^. In this study, it served as a training set, with the evaluation (test set), being done based on the image data described in the methods section (study subjects). The neural network architecture chosen was a U-Net, with three encoding and decoding layers. Categorical cross-entropy was used as the loss function and models and model parameters were evaluated based on a leave one subject out cross validation approach using the Auckland data set^45^. The pre-processing involved denoising and flat fielding of the image data to account for noise and intensity inhomogeneity, respectively, and the post-processing thresholds skeletonize the output nerve probability map from the neural network to generate the final nerve fiber segmentation. This enables reporting of CNFL (mm/mm^2^) and CNFrD. Analyzed image examples by Voxeleron deepNerve are shown in Fig. 4C,F,I,L.

### Statistical analysis

Prism (version 8.4.3 for Mac, GraphPad software Inc., CA, USA) and MatLab (version v2019b for Windows, Mathworks Inc., USA) were used for the statistical analyses and graphic illustrations. A Shapiro–Wilk test was used to assess data for normality (*P* < 0.05). Significant deviations from normality were not observed. We used an unpaired *t*-test or one-way analysis of variance (Post-hoc Sidak’s test) to compare the results between the MS and healthy controls groups and between the CIS, RRMS, SPMS groups and healthy controls respectively. A two-sided *P* value was favored on the assumption of unequal population means and a *P* < 0.05 was considered significant. Spearman correlations and the ICC were calculated to assess the relationship between ACCMetrics and Voxeleron deepNerve; secondary analyses adjusted Spearman correlation for age, sex, and race. Spearman correlations were adjusted using methods described by Liu et al.^46^ Agreement between the two analysis methods was assessed by means of Bland–Altman plots (average vs. the difference between values) and calculating the upper and lower limits of agreement. We also considered Bland Altman plots that additionally modeled the variability in the difference as a function of the level of measurement that allow for more complex associations between measures^30^ (e.g., we regressed the difference between measures as a function of the level of measurement that can account for differences in the SD across the two measures). The ICC was estimated using a linear mixed effects model with a random effect to estimate within and between person error (for two measures per person), following methods originally described by Shrout et al.^47^ Generally, a higher ICC with a lower limit of the 95% CI ≥ 0.75 indicates excellent reproducibility^48^.
